# Efficacy in treatment of subclinical cervical HPV infection without intraepithelial neoplasia: systematic review

**DOI:** 10.1590/S1516-31802000000400007

**Published:** 2000-07-07

**Authors:** Fábio Russomano, Aldo Reis, Maria José de Camargo, Maria Virgínia Peixoto Dutra, Sandra Costa Fonseca, Jean Anderson

**Keywords:** HPV, Cervical Intraepithelial Neoplasia, Treatment, Systematic Review, HPV, Neoplasia Intraepitelial Cervical, Tratamento, Revisão sistemática

## Abstract

**CONTEXT:**

The treatment of the subclinical Human papillomavirus (HPV) infection of the uterine cervix is controversial.

**OBJECTIVE:**

To assess the efficacy of any therapy for subclinical HPV infection of the cervix without intraepithelial neoplasia, via a search in the medical literature.

**METHOD:**

We performed a systematic review with a comprehensive reference search in Medline, LILACS, Excerpta Medica, AIDSLINE, Popline, Cochrane Library and other authors’ reference lists to identify experimental studies of therapy for subclinical HPV infection without intraepithelial neoplasia of the uterine cervix. In order to identify unpublished studies, we also contacted experts in the area, clinical trial registries, pharmaceutical industries, government and research institutions. We also searched on the Internet and in the book-of- abstracts of some medical conferences. The studies identified were masked and selected by inclusion criteria to help ascertain their internal validity. The data about regression or progression of HPV infection were extracted from the studies included.

**RESULTS:**

We identified 67 studies related to the treatment of sub- clinical HPV infection without intraepithelial neoplasia of the uterine cervix. Only five clinical trials matched the inclusion criteria and none demonstrated significant differences between the experimental group and the control group concerning regression of HPV infection (with or without CIN I) or progression to higher grades of CIN.

**CONCLUSION:**

The evidence we found in the medical literature regarding the efficacy of any therapy for subclinical HPV infection without intraepithelial neoplasia of the uterine cervix was unsatisfactory.

## RESUMO

**CONTEXTO:**

O tratamento da infecção subclínica pelo papilomavírus humano (HPV) no colo uterino é controverso.

**OBJETIVO:**

Avaliar a eficácia de alguma terapia para infecção pelo HPV no colo uterino sem neoplasia intraepitelial.

**MÉTODO:**

Realizamos uma revisão sistemática com uma busca abrangente de estudos no Medline, LILACS, Excerpta medica, AIDSLINE, Popline, Cochrane Library e listas de referências de outros autores para identificar estudos experimentais sobre terapia para a infecção subclínica pelo HPV sem neoplasia intraepitelial no colo uterino. Para identificar artigos não publicados, também contactamos especialistas da área, registros de ensaios clínicos, indústrias farmacêuticas, instituições governamentais e de fomento à pesquisa. Também procuramos por estudos através da Internet e em livros de abstracts de alguns eventos médicos. Os estudos identificados foram mascarados e selecionados por critérios de inclusão que auxiliaram a garantir sua validade interna. Os dados sobre regressão ou progressão da infecção pelo HPV foram extraídos dos estudos incluídos.

**RESULTADOS:**

Identificamos 67 estudos relacionados ao tratamento da infecção subclínica pelo HPV sem neoplasia intraepitelial do colo uterino (NIC). Apenas cinco ensaios clínicos atenderam aos critérios de inclusão e nenhum demonstrou diferenças significativas entre o grupo em teste e o grupo-controle no que se refere a regressão da infecção pelo HPV (com ou sem NIC I) ou progressão para graus mais altos de NIC.

**CONCLUSÃO:**

As evidências de eficácia de alguma terapia para infecção subclínica pelo HPV sem neoplasia intraepitelial no colo uterino encontradas na literatura médica foram insatisfatórias.

## INTRODUCTION

The treatment of subclinical human papillomavirus (HPV)^1^ infection is a matter of controversy. As defined by Reid et al (1982), in this kind of HPV infection, instead of producing an overt condyloma, a "macroscopic invisible, non-papilliferous epithelial hyperplasia" can be produced. Considering the high incidence of cervical cancer worldwide, it would be reasonable to try to treat all forms of HPV infection to prevent future cancer development. However, considering the high frequency of spontaneous regression of HPV infection and the lack of a specific antiviral drug, many experts follow these patients conservatively and treat cervical intraepithelial neoplasia (CIN), should it develop.

The goal of this paper is to search for evidence of efficacy of any treatment for subclinical HPV infection of the cervix when there is no concomitant CIN using a systematic review of the medical literature.

## METHODS

### Study search

A comprehensive search in the medical literature was performed, including both published and unpublished studies. Studies were identified using reference databases (Medline, Compact Medical Li- brarian-AIDSLINE, LILACS, Excerpta Medica, Popline and the Cochrane Library), other authors’ reference lists, abstracts of studies presented in meetings related to cervical pathology, information from experts that work in the area, information from institutions that financially support clinical research, Clinical Trils Registries, government agencies, pharmaceutical companies and Internet sites.

In digital media, the search was carried out using terms related to uterine cervix, treatment and subclinical HPV infection (Table 1). The search period ranged from 1977 to March 1997, since the first studies linking HPV to cervical neoplasia date from 1977. Studies published in English, Spanish or Portuguese were included.

**Table 1:** The strategy used to search for related studies in Medline (applies only to Silverplatter 3.1)

1:	WART* in TI, AB
2:	CONDYLOM* in TI, AB
3:	PAPILLOMA* in TI, AB
4:	HPV in TI, AB
5:	INTRAEPITHELIAL in TI, AB
6:	KOILOCYT* in TI, AB
7:	"ABNORMAL CERVICAL SMEARS" in TI, AB
8:	"ABNORMAL SMEARS" in TI, AB
9:	explode PAPILLOMA
10:	#1 or #2 or #3 or #4 or #5 or #6 or #7 or #8 or #9
11:	THERAP* in TI, AB
12:	TREAT* in TI, AB
13:	MANAGEMENT in TI, AB
14:	explode TREATMENT
15:	#11 or #12 or #13 or #14
16:	CERVI* in TI, AB
17:	GENITAL in TI, AB
18:	#16 or #17
19:	tg=FEMALE
20:	#10 and #15 and #18 and #19
21:	pt=REVIEW
22:	pt=MEETING REPORT
23:	pt=LETTER
24:	pt=COMMENT
25:	pt=EDITORIAL
26:	pt=GUIDELINE
27:	#21 or #22 or #23 or #24 or #25 or #26
28:	#20 not #27.

The studies identified were obtained and their methodology was assessed to verify their internal validity.

The criteria used to diagnose HPV infection varied between authors. Different diagnostic criteria were accepted if they were used consistently within a given study, i.e. used to both select patients and to determine outcome. This allowed us to include studies done prior to the availability of HPV-DNA detection methods and to avoid selection bias.

HPV infection diagnostic criteria included cytology, colposcopy, histology or a combination of these methods. Some of the more recent studies also used HPV-DNA detection methods in their analysis.

An instrument was built to assess the internal validity of each study and identify studies for selection, based on criteria adapted from Guyatt et al^2-4^ (Table 2). We assessed the reliability of this instrument between two of the present authors (FR & SCF), assessing the internal validity of a sample of 15% of the studies identified, and concluding that it had good reliability.^5-8^

**Table 2:** Inclusion criteria (adapted from Guyatt et al 1993)

**GENERAL ASPECTS**
Study of treatment for subclinical HPV infection of the cervix without intraepithelial neoplasia (studies using the term "low grade lesion" published after 1990, were also considered). Complete investigation, with final results shown.
**METHODOLOGICAL ASPECTS**
Randomized clinical trial, placebo-controlled. Identical and valid diagnostic methods used to identify infected women and to measure the results after intervention. Loss to follow-up of 10% or less. Follow-up period of at least one month. Intention-to-treat analysis. Experimental and control groups similar at entry into the study concerning age and other factors that could interact with any treatment modality. Differences need to have been checked prior to analysis. The groups must be treated and followed equally, apart from the experimental intervention.

Each study was masked, with only its methodology section being assessable. Studies that met inclusion criteria had their data extracted, including sample size, treatment tested and treatment outcome in the experimental and control groups. Data was analyzed and synthesized but could not be combined because of differences in treatment modalities.

## RESULTS

### Results of the study search

Studies identified are listed in the Reference section (references 9 through 75) and a summary of the results of the study search from each source is shown in Table 3. Eight studies could not be obtained for review (references 9 through 16).

**Table 3:** Reference sources and their results (number of studies identified)

Reference sources* # o	f identified studies	# of non-obtained studies
Medline (alone)	17	2
Medline & Excerpta Medica	14	–
Medline & LILACS	4	1
Medline & other authors’ reference lists	10	–
Medline, Excerpta Medica & other authors’ reference lists	4	–
Excerpta Medica (alone)	3	1
Excerpta Medica & other authors’ reference lists	2	–
LILACS (alone)	1	–
LILACS & other authors’ reference lists	2	–
LILACS & experts’ information	1	–
Other authors’ reference lists (alone)	7	2
Abstracts of studies presented in medical meetings	2	2
**Totals**	**67**	**8**

* Reference sources that were used but did not identify studies include: Compact Library AIDS, Cochrane Library, Popline, Internet & electronic journals, Clinical Trials Registries, government institutions, research sponsoring institutions, pharmaceutical companies.

### Study selection

Of the 59 studies obtained in their full version, 5 were included^19-23^ and 54 were excluded.^17,18,24-75^ The primary reason why studies were excluded was their study design (42 studies - 77%). The remaining 12 (22%) showed one of the following situations: four were clinical trials but were not placebo-controlled; two had more than 10% of patients lost to follow-up; one tested adjuvant therapy instead of primary therapy; one assessed the outcome differently in experimental and control groups; one analyzed patients with HPV without CIN together with patients with CIN I (published before 1990) or CIN II; one tested the therapy on clinically-expressed HPV infection; one reported only preliminary data; and in one we were not able to extract the information needed from it.

In Table 4 and in the following text we show a brief summary of the five studies that met the inclusion criteria. Two studies included at first were then excluded. The first one was the study by Yliskoski et al, 1991.^17^ They had studied a group of patients with HPV without CIN together with others with CIN. We wrote to one of the authors (KS) and he told us that the data was not available anymore. The other study was the one from Woodman et al, 1993.^18^ This study presents treatment outcomes from patients with progression risk and it was not stated how many patients had regression or progression. We wrote to the first author but we have not had any reply yet.

**Table 4:** Included studies

Author, publication year	Patients selected	Treatment method tested
Boothby et al.,^19^ 1990.	HPV infection without CIN	50% trichloroacetic acid application.
Diakomanolis et al.,^20^ 1990.	HPV infection without CIN	CO_2_ laser vaporization of the entire transformation zone, distal region of the endocervical canal and, more superficially, the ectocervical region outside the transformation zone.
Ruge et al.,^21^ 1992.	HPV infection with or without CIN I	CO_2_ laser vaporization of the entire transformation zone and adjacent areas to 4-5 mm in depth.
Hording et al.,^22^ 1993.	HPV infection with or without CIN I	CO_2_ laser vaporization of the entire transformation zone.
Fairley et al.,^23^ 1996.	HPV infection without CIN*	Beta-carotene, 30 mg/day, taken orally for one year.

* Other groups were selected, with CIN I to CIN III, but these data were not used in our review.

### Boothby et al.,^19^ 1990.

This is a randomized clinical trial testing trichloroacetic acid (TCA) at 50% concentration, applied to the cervix. Thirty-four women with biopsy-proven HPV infection of the cervix were randomly allocated to receive TCA or normal saline in a blinded fashion. The outcome was assessed 16 weeks after treatment. The treatment was considered a failure if there was evidence of HPV on Pap smear or biopsy. The text does not provide a similarities analysis between the groups after treatment allocation. Although the authors state that patients and investigators were blinded to the treatment allocation, there is a marked difference in cervix appearance after applying the two substances. The application of TCA turns the cervix surface white, as a result of chemical coagulation of its epithelium proteins and impairment in viewing the color of the stroma. If this had been reported in patient records, to the patient or kept in mind by the medical doctor, the blinding could have been impaired (potential source of assessment bias). When the first 15 patients in each group were analyzed, no differences were noted and the study was closed. No patients were lost to follow-up. Six women in the experimental group and two in the control group had undesirable side effects. Two women in the experimental group developed CIN I.

### Diakomanolis et al.,^20^ 1990.

This study enrolled 452 women with biopsyproven HPV cervical infection without CIN. The authors report that "patients were allocated randomly under a specific number" (page 506), which is unclear to us. There is no similarities analysis between the study groups after the allocation for treatment. The study group had vaporization of the entire transformation zone, distal endocervical canal and "brushing" of the cervix portio while the control group received no treatment. It was not possible to blind the procedure being studied to patients and investigators. After 18 months, 8 (5.6%) women from the treatment group and 24 (7.7%) of the control group showed progression to pre- invasive disease (P = 0.42). No data was presented on the disappearance of the disease.

### Ruge et al.,^21^ 1992.

In this study 50 women with biopsy-proven HPV infection with or without concomitant CIN I were randomly allocated to a laser group or a control group. There is no information about the randomization method used. However, despite this limitation, there were no statistical differences between study and control groups in age, smoking, socioeconomic status, sexual habits and contraceptive methods. The treatment group had their entire transformation zone "evaporated" to a depth of 4-5 mm. The control group had no treatment. As in the previous study, there was no patient or investigator blinding regarding which group each patient was allocated to. After 12 months, the authors analyzed regression, persistence and progression and found no statistical differences between the two groups.

### Hording et al.,^22^ 1993.

In this study, the authors analyzed 46 women with biopsy-proven HPV cervical infection with or without CIN I. These women were allocated randomly to a treatment group (laser vaporization of the cervix) or to a control group without treatment. The authors did not report the randomization method used and there is no analysis of group similarities after treatment allocation. As in the two previous studies, there was no patient or investigator blinding concerning treatment or control procedures. No patients were lost to follow-up, with mean follow-up of 28 months (range of 12 to 54 months). There were no significant differences between the study groups.

### Fairley et al.,^23^ 1996.

Considering epidemiological and laboratory evidence that beta carotene may be of value in the treatment of pre-invasive cervical disease, these authors performed a double-blind clinical trial in which 117 women were randomly allocated to receive beta carotene (30 mg/daily) or placebo (lecithin, 400 mg/daily) for one year. There is no report of the randomization method used but the groups were similar in age, marital status, smoking, contraceptive method, parity and number of sexual partners. After one year of follow-up no differences were found between the study groups in terms of lesion clearance. Thirteen per cent of the women receiving betacarotene noticed yellowing of the skin.

### Information extracted from the selected studies

Table 5 shows data extracted from the selected studies concerning progression of HPV infection without CIN to presence of CIN or from HPV with CIN I to higher grades of CIN. Table 6 shows data related to disappearance of subclinical HPV with or without CIN I.

**Table 5:** Progression of HPV infection with or without CIN I

Author, year	Experimental group (n)	Progression n (%)	Control group (n)	Progression n (%)	P-value	Follow-up
Diakomanolis et al.,^20^ 1990	142	8 (5.6%) HPV to CIN I-III	310	24 (7.7%) HPV to CIN I-III	0.42	18 months
Ruge et al.,^21^ 1992	25	2 (8%) HPV-CIN I to CIN III	25	2 (8%) HPV-CIN I to CIN III	0.60	12 months

**Table 6:** Regression of HPV infection with or without CIN I

Author, year	Experimental group (n)	Disappearance or regression of HPV subclinical lesionsn (%)	Control group (n)	Disappearance or regression of HPV subclinical lesions n(%)	P-value	Follow-up
Boothby et al.,^19^ 1990	15	1 (6.7%)	15	3 (20%)	0.52	4 months
Ruge et al.,^21^ 1992	25	21 (84%)	25	19 (76%)	0.48	12 months
Hording et al.,^22^ 1993	23	21 (91.3%)	23	16 (69.5%)	0.11	12 to 54 months
Fairley et al.,^23^ 1996	33	20 (60.6%)	36	22 (61.1%)	0.96	12 months

Three studies included an analysis of the presence of HPV-DNA and its disappearance after the experimental period. These data are shown in Table 7.

**Table 7:** HPV-DNA detection in the selected studies

Author, year	HPV-DNA analysis
Ruge et al.,^21^ 1992	HPV-16 DNA detected by PCR, before and after the study period. There was no difference between the success rate of the HPV+ laser treatment group (78.5%) compared to the control group (80%) in achieving HPV-16-DNA disappearance.
Hording et al.,^22^ 1993	HPV 6,11,16,18 and 33 detected by PCR before and after the study period. There was a significant statistical difference between the HPV+ laser treatment group (12/13, 92%) and control group (2/7, 29%, p = 0.007).
Fairley et al.,^23^ 1996	HPV-DNA detected by PCR or Hybrid Capture. There was no difference in the detection of HPV-DNA between treatment and control groups.

## DISCUSSION

From the 59 studies identified, obtained and reviewed, only five met the inclusion criteria. None of these found any difference between treatment and control groups in either progression or regression of subclinical HPV infection of the cervix. However, these studies were limited by one or more problems, such as the sample size, difficulties in blinding patients and investigators, and we do not know the randomization method used by four of them. These limitations leave us unsure that the treatments tested are ineffective.

Most studies identified were excluded because of lack of internal validity for adequately testing any form of treatment.

Although we could not obtain 8 studies in their full version, even after attempts using national or foreign libraries and asking the authors for them (in the case of studies identified in Books of Abstracts from medical meetings), assessment of their abstracts allowed us to conclude that they would probably be excluded because they were not clinical trials.

In the decision-making process concerning the use of any therapy to treat this kind of HPV infection, we must consider the lack of evidence that treatment is better than no treatment. The use of therapies in which efficacy is not proven can lead to unnecessary waste of money or can be worse than the illness itself, considering the physical consequences of some of them and the psychological consequences of trying to treat an infection that can present recurrence.

Well-conducted randomized clinical trials can be expected to prove some therapy to be effective against subclinical HPV infection, preferably with patient and investigators being blinded, and with sufficient sample size with enough power to avoid beta error (i.e. saying that there is no difference between treatment and no treatment when it actually exists and the only reason that statistical significance was not achieved was because of the small sample size).

However, applying financial resources to conduct an experiment to clarify this question appear unreason-able to us. Given the high prevalence reported by some authors, varying between 13 to 20% in developing countries, in which Brazil is included,^76^ and the transient characteristic of most of these infections,^77^ the cost of medicine usage, therapeutic procedures or vaccines would not be reasonable in the prevention of cervical cancer.

On the other hand, there is already strong evidence of efficacy in treatment of pre-invasive diseases in order to prevent cervical cancer. It seems to us that it is more cost-effective to apply these procedures to the patients that present CIN II/III, considering that they are a small proportion of all HPV-infected women.

## CONCLUSIONS

The evidence we found for efficacy in treatment of subclinical HPV infection of the cervix without CIN was unsatisfactory. However, considering the limitations of the selected studies, particularly in terms of sample size, possible efficacy in these treatments cannot be excluded.

In many of the studies excluded for methodological reasons, the authors’ conclusion was clearly in favor of the treatments tested, possibly leading to ineffective or even potentially harmful therapies and to unnecessary costs.

Medline, LILACS and Excerpta Medica were the most useful sources of references, and identified all of the studies included.
